# Building trust in the integration of artificial intelligence into chemical risk assessment: findings from the 2024 ECETOC workshop

**DOI:** 10.1007/s00204-025-04286-8

**Published:** 2026-02-17

**Authors:** Timothy W. Gant, Alistair Boxall, Daniel Burgwinkel, Maryam Zare Jeddi, Ivo Djidrovski, Steffi Friedrichs, Barry Hardy, Thomas Hartung, Daniela Holland, Andreas Karwath, Anne Kienhuis, Nicole Kleinstreuer, Zhoumeng Lin, Emma L. Marczylo, Antonino Marvuglia, Hua Qian, Bennard van Ravenzwaay, Paul Rees, Haralambos Sarimveis, Tewes Tralau, Lucy Wilmot, Adam Zalewski, David Rouquié

**Affiliations:** 1 Centre for Radiation, Chemical and Environmental Hazards, Toxicology Department, UK Health Security Agency, Harwell Science Campus, Didcot, OX11 0RQ UK; 2 School of Public Health, Imperial College, London, UK; 3 Department of Environment and Geography, University of York, York, UK; 4 Edelweiss Connect GmbH, Basel, CH Switzerland; 5 Shell Global Solutions International B.V, The Hague, Netherlands; 6 Institute for Risk Assessment Sciences (IRAS), Utrecht University, Utrecht, Netherlands; 7 AcumenIST SRL, Brussels, Belgium; 8 Center for Alternatives to Animal Testing (CAAT), Doerenkam-Zbinden-Chair for Evidence-Based Toxicology, Bloomberg School of Public Health, Johns Hopkins University, Baltimore, MD US; 9 University of Konstanz, CAAT Europe, Konstanz, Germany; 10 ExxonMobil Petroleum and Chemical BV, Machelen, Belgium; 11 College of Medicine and Health, School of Medical Sciences, University of Birmingham, Birmingham, UK; 12 Department of Innovative Testing Strategies, Centre for Health Protection, National Institute for Public Health and the Environment (RIVM), Bilthoven, Netherlands; 13 Facutly of Veterinary Medicine, Department of Population Health Sciences, Insitute for Risk Assessment Sciences, Utrecht University, Utrecht, Netherlands; 14 Office of the Director (OD), Division of Program Coordination, Planning, and Strategic Initiatives, National Institutes of Health (NIH), Maryland, US; 15 Department of Environmental and Global Health, College of Public Health and Health Professions, University of Florida, Gainesville, FL US; 16 Center for Environmental and Human Toxicology, University of Florida, Gainesville, FL US; 17 Luxembourg Institute of Science and Technology (LIST), Esch Sur Alzette, Luxembourg; 18 ExxonMobil Biomedical Sciences, Inc, Spring, TX US; 19 Division of Toxicology, Wageningen University, Wageningen, Netherlands; 20 Department of Biomedical Engineering, Swansea University, Bay Campus, Swansea, SA18EN UK; 21 School of Chemical Engineering, National Technical University of Athens, Athens, Greece; 22 German Federal Institute for Risk Assessment (BfR), Berlin, Germany; 23 ECETOC, Rue Belliard 40, 1040 Bruxelles, Belgium; 24 Pharmaceuticals, Preclinical Development, Bayer Research & Development, Berlin, Germany; 25 Bayer SAS, Bayer Crop Science, Sophia-Antipolis, France

**Keywords:** AI, Toxicology, Hazard and risk assessment, FAIR data, Trust, Explainable AI

## Abstract

Artificial Intelligence (AI) is increasingly influencing chemical risk assessment, enabling faster, more comprehensive, and potentially more ethical assessments. The application of AI in chemical risk assessment refers to both generative and predictive algorithms encompassing machine learning, to analyse complex chemical, biological, and environmental data and provide insights into adverse effect potential for humans and ecosystems. AI systems support the prediction of chemical hazards, exposure levels, and adverse effects by learning from experimental results, mechanistic models, and regulatory datasets, thereby enhancing the efficiency of safety evaluations.

In October 2024, ECETOC held an international workshop, with experts from academia, industry, and regulatory bodies, to reflect upon the historical challenges in integrating multidimensional omics technologies into chemical regulation and explore the current capabilities and future potential of AI in toxicology and regulatory science. Discussions emphasised that implementation of Findable, Accessible, Interoperable, and Reusable (FAIR) data principles is not just a best practice but rather a prerequisite for building transparent, reliable, and unbiased AI systems. The reliability of AI in producing scientifically valid and socially responsible outcomes depends fundamentally on the availability of FAIR data. However, ensuring trustworthiness also requires robust governance frameworks that go beyond data and human oversight. Critical enablers of responsible AI in chemical risk assessment are rigorous governance, explainability, fit-for-purpose applications, and human oversight. ECETOC supports the development of flexible and iterative frameworks advancing development, validation, transparency, accountability, and trust in AI applications in chemicals regulation.

## Highlights

AI enhances but does not replace human judgment in risk assessment.

Generative and predictive AI offer complementary functions in toxicology and risk assessment.

FAIR data principles are critical for transparency and reproducibility.

Governance frameworks must evolve rapidly to keep pace with AI development, particularly for generative models.

Lessons from Omics data analysis and regulatory acceptance can inform responsible AI integration and acceptance.

Harmonized international frameworks are urgently needed to support development, validation, transparency, and trust in AI applications.

## Introduction

Artificial Intelligence (AI) can mimic human cognitive processes by analysing data to derive new knowledge, learn from patterns, and predict or generate outputs. Unlike biological intelligence (BI), which incorporates emotional responses and evolved instincts, AI operates via machines through logical, data-driven processing.^1^ While AI lacks emotional and perceptual dimensions intrinsic to BI, and is fundamentally dependent on the data used to train the models, it offers significant advantages in speed, scalability, and complex, large-scale data handling capacity. Therefore, AI should be viewed as a technology able to process large and diverse datasets rapidly, while human oversight ensures ethical and interpretative judgment, i.e., Augmented Intelligence. This hybrid approach is particularly critical for fields involving uncertainty, such as hazard identification, exposure assessment, and risk communication.

In BI, the outcomes of past experiences form the foundation for analysing new information. When certain experiences prove beneficial for survival, they are gradually reinforced through evolutionary selection, eventually becoming encoded in the genome as instinct. AI follows a conceptually similar process: it learns from data and builds models that reflect accumulated experience. Like BI, AI can adapt and generate new insights based on what it has learned, enabling it to make predictions and solve novel problems.

AI can act like BI in that it both analyses data to generate an output and derives new data. The latter can, for example, be equated in BI to the production of a new image or piece of writing. These two modes of operation are called predictive and generative. Both predictive and generative AI use core algorithmic categories (e.g., machine learning, deep learning, optimization, and natural language processing). These algorithms identify variable patterns from training data sets and use these to define the variable weightings within the algorithms that comprise the model. In chemical risk assessment, specifically in toxicology, AI offers profound potential to enhance human-led assessment, reduce animal testing, accelerate hazard identification, and support evidence-based regulatory assessments (Hartung 2023; Hartung and Kleinstreuer 2025; Kleinstreuer and Hartung 2024). AI is no longer just a future promise in chemical risk assessment—it is actively transforming current practices. From predictive modelling to generative data synthesis, AI is reshaping how we interpret complex datasets, identify hazards, and make regulatory decisions. Its integration into workflows is accelerating the shift toward more efficient, ethical, and mechanistically informed approaches, such as those seen in next generation risk assessment (NGRA) and new approach methodologies (NAMs). In all the hitherto accepted applications of AI in risk assessment, however, AI remains limited to the data processing steps, while the decision-making based on AI-processed data currently remains a human capacity, rendering AI to the abovementioned ‘augmented intelligence’ role.

**Fig. 1:**
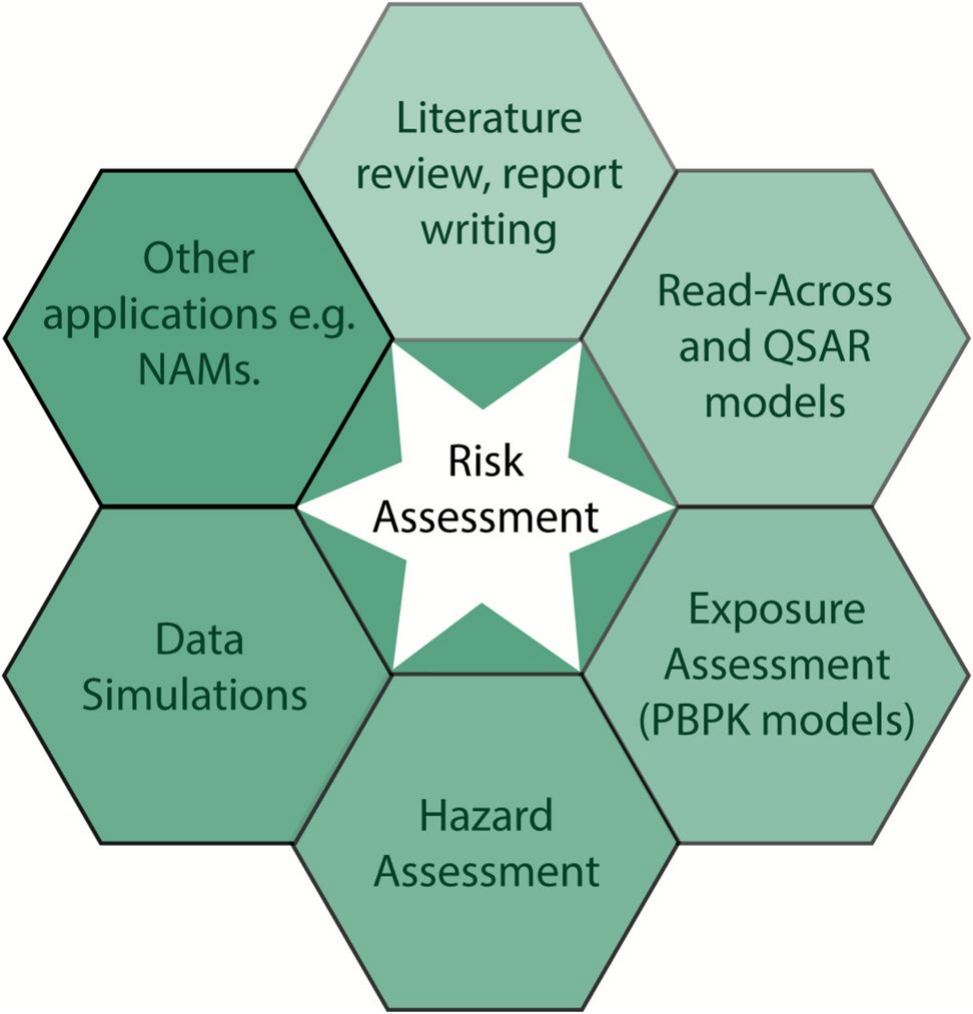
Schematic illustration of the potential areas of AI application in chemical risk assessment

In October 2024, ECETOC, held an international workshop, with experts from academia, industry, government and regulatory bodies exploring the current capabilities and future potential of AI in toxicology and regulatory science. This ECETOC workshop explored the reality of AI in chemical risk assessment where several areas for potential application have been identified (Fig. 1).

## Workshop objectives and format

The ECETOC AI workshop took place over two days, both online and in person, at the Bayer Crop Science facility in Sophia Antipolis France in October 2024. The workshop was attended by 30 in person participants with over 100 participants joining online. The objectives were:

- To understand the scope and types of AI relevant to chemical risk assessment.
- To assess the practical and regulatory challenges of applying AI tools.
- To identify key areas for future policy and technological development.

Day 1 featured expert presentations on foundational AI concepts, real-world applications, and barriers to regulatory acceptance. Day 2 included three in-person breakout sessions focusing on data challenges, practical applications, and trust-building mechanisms.

This manuscript aims to chart a path forward based on the insights gained during this informative two-day workshop, recognising that the AI field is rapidly evolving, which makes it essential for all stakeholders to agree on a minimum common ground of mutual understanding and shared practices.

## AI subtypes and applications in toxicology

**Fig. 2:**
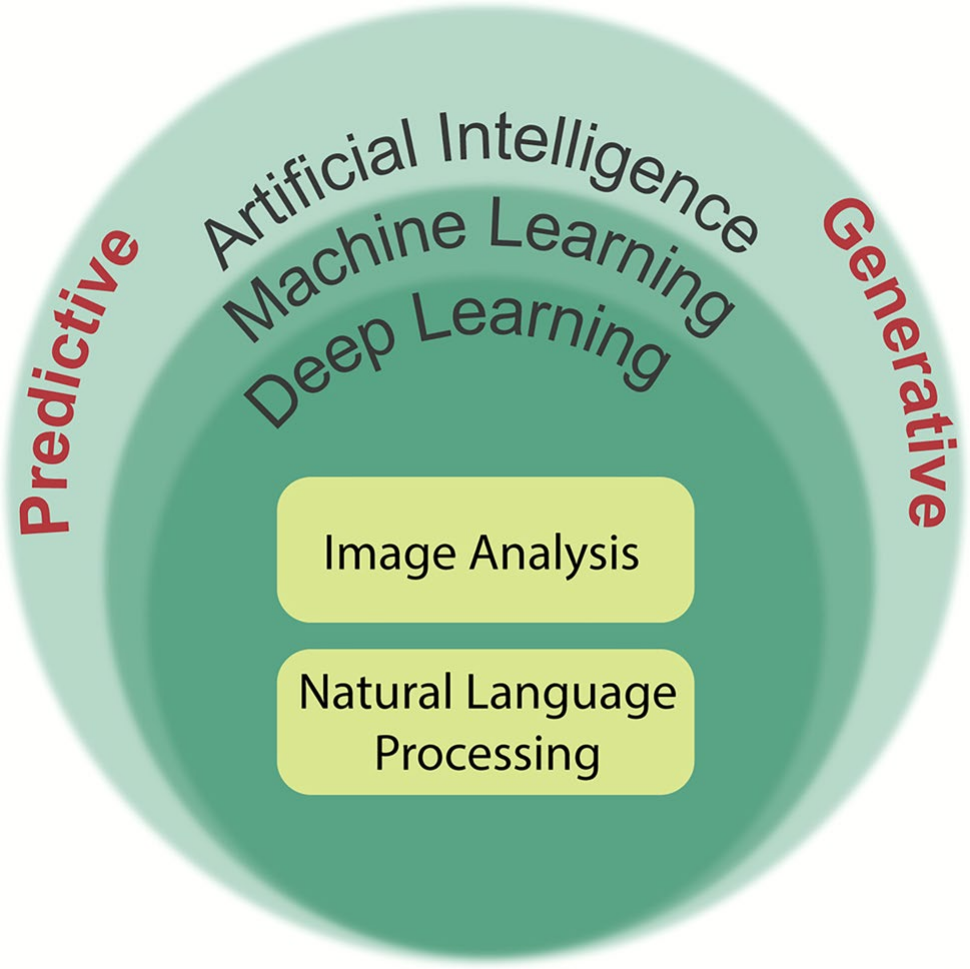
Illustration of the main subdivisions of AI. [Acknowledging that various interpretations exist, this figure provides a simplified introductory perspective]

AI encompasses a range of computational techniques that enable systems to perform tasks typically requiring human intelligence. This includes **machine learning**, which allows systems to learn from data without being explicitly programmed, and **generative AI** (Fig. 2), which can create new content such as text, images, or designs. A key subset of machine learning is **deep learning**, often implemented using neural networks, which excels at identifying patterns in large, multimodal, and unstructured datasets such as images, audio, and natural language.

### Predictive AI

Predictive AI is an application of machine learning that uses existing data to train models that can forecast outcomes or classify new inputs. This includes supervised learning methods (e.g., random forests, support vector machines, neural networks) and unsupervised methods (e.g., clustering). The algorithms are trained on a dataset, and re-training can occur until a point is reached where the model works well enough with sufficient training data to accurately analyse test data. A thorough evaluation is necessary to determine whether the model is being applied to data that significantly differs from its training set. External validation may increase confidence in the model, or show areas in which its predictions may become unreliable or inaccurate, thereby defining the applicability domain.

Applications of predictive AI in chemical risk assessment include:

- Classifying chemical absorption, distribution, metabolism, excretion (ADME), and toxicity properties via quantitative structure activity relationship (QSAR) models;
- Classifying in vitro bioactivity patterns (e.g., transcriptomic, cell painting);
- Identifying toxicological key events or endpoint responses from imaging.

### Generative AI

Generative AI refers to the creation of new data that mimics the statistical properties of a training dataset, without being produced through direct experimentation or physical processes. One of the major achievements in this area is Large Language Models that power almost all the chatbots such as ChatGPT. Among the other advanced techniques in this area are **Generative Adversarial Networks (GANs)**, which consist of two interacting components: a **generator** and a **discriminator**.

**Fig. 3:**
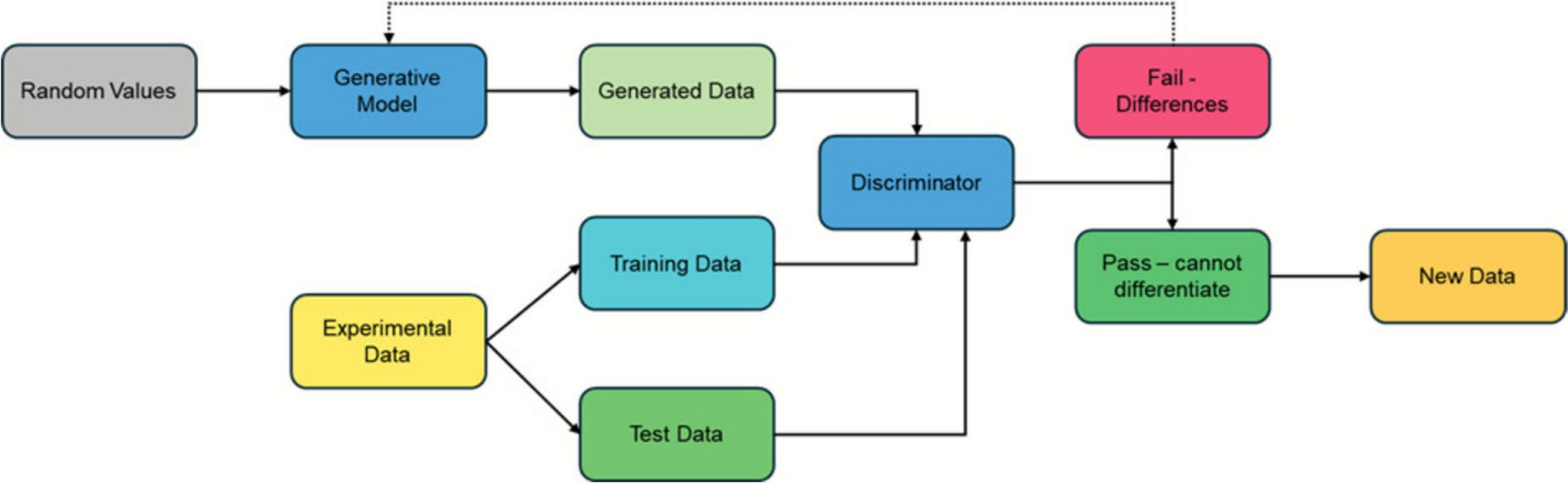
Flowchart indicating the training and workflow within a generative adversarial network (GAN) training and workflow

The **generator** is a neural network trained to produce synthetic data by transforming random input (often noise) into outputs that resemble the training data. The **discriminator**, also a neural network, evaluates whether a given data instance is real (from the training set) or generated (from the generator). It has access to the same class of real data as the training set and provides feedback to improve the generator’s performance. The process is adversarial: the generator aims to create data that is increasingly indistinguishable from the real data, while the discriminator attempts to detect synthetic outputs. Through iterative training, both networks improve—the generator becomes better at “fooling” the discriminator, and the discriminator becomes better at identifying real versus generated data. This dynamic leads to the production of **high-fidelity synthetic data** that closely resembles real-world observations. This process can be used to produce new text, images, numerical data or new molecules (Fig. 3).

Examples presented at the workshop for generative AI in toxicology included:

- Compound de novo design with high probability to induce specific biological responses;
- AnimalGAN (Chen et al. 2023) which replicates clinical biochemistry profiles of rats across substances and doses;
- ToxGAN (Chen et al. 2022) which generates synthetic gene expression profiles, useful for early-stage compound/substance screening.

These models offer opportunities for reducing animal testing and extending in silico assessment, but require strict validation and provenance controls.

The generative model for de novo chemical design aims to bridge systems biology and molecular design, enabling the automated creation of molecules with a high likelihood of inducing a desired transcriptomic or cell painting profile. A proof of concept demonstrated the feasibility of this approach using large public datasets from transcriptomics and cell painting. However, significant work remains to accurately identify in vitro patterns that are causally linked to adverse outcomes in humans. This is essential to guide the design of chemicals that avoid such undesirable properties while retaining the necessary pharmacological properties for drugs or biological effects in the target species for crop protection products. Overall, this work represents a promising step toward the paradigm of AI-guided chemical risk by design (Marin Zapata et al. 2023; Méndez-Lucio et al. 2020).

The AnimalGAN and the ToxGAN models are two GAN models trained on the TG-GATEs dataset. The TG-GATEs dataset consists of pathology, biochemical and gene expression data for 170 substances generated in vivo in rats and in vitro in cultured rat and human primary cells (Igarashi et al. 2015). For AnimalGAN, an example was presented where rat clinical pathology data from 138 substances was selected from the TG-GATEs dataset (Chen et al. 2023). This was then divided into two sets: the training set of 110 substances representing data from 6442 rats to generate an AnimalGAN model; and a test set of 28 substances were used to evaluate the model. The AnimalGAN model was used to generate new data for a further 28 substances taken from another database (DrugMatrix^2^) and the data generated was indistinguishable from the real data derived experimentally.

**Fig. 4:**
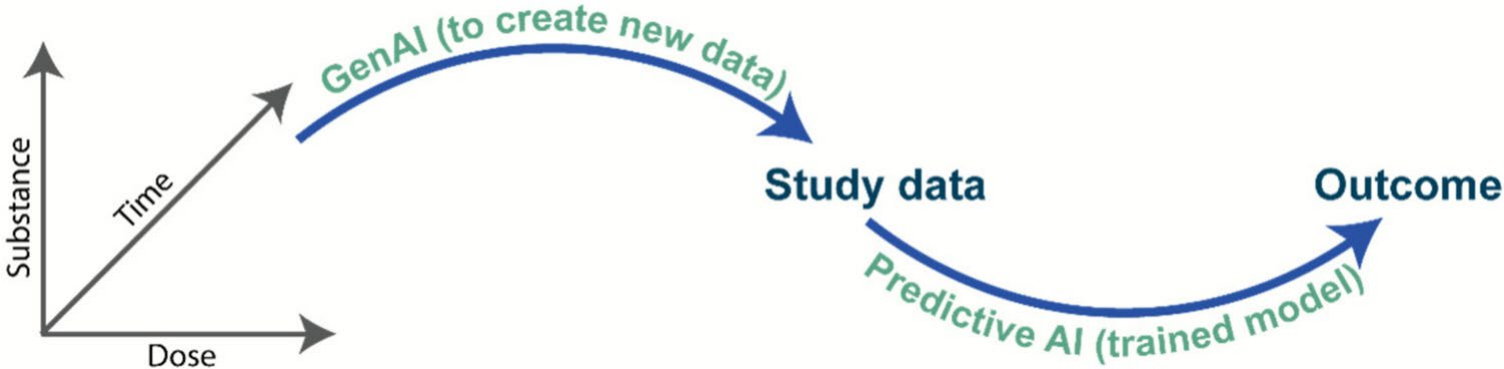
Illustration of the derivation of new data on hazard from a trained GAN mode as a function of a chemical substance, its dose and time of exposure

This type of model opens the possibilities of extending experimentally-derived datasets or generating new ones based on pieces of derived information such as structural or gene expression similarity (Fig. 4). The ToxGAN model used the same principles but different data: gene expression data (Chen et al. 2023, 2022; Li et al. 2024a). For novel molecules there is the possibility of using a QSAR analysis to match to a similar molecule then using the generative prompt of substance, dose and time of exposure to generate an entirely new clinical chemistry or gene expression profile for the novel substance.

### Applications

Another—more intuitive—way of looking at AI is to subdivide by application domain; three domains dominate current AI applications:

- **Image analysis**: automated scoring of histology or high-content screening;
- **Natural language processing**: information extraction from scientific literature;
- **Data mining & modelling**: multi-omics integration, pattern mining, clustering.

**Fig. 5:**
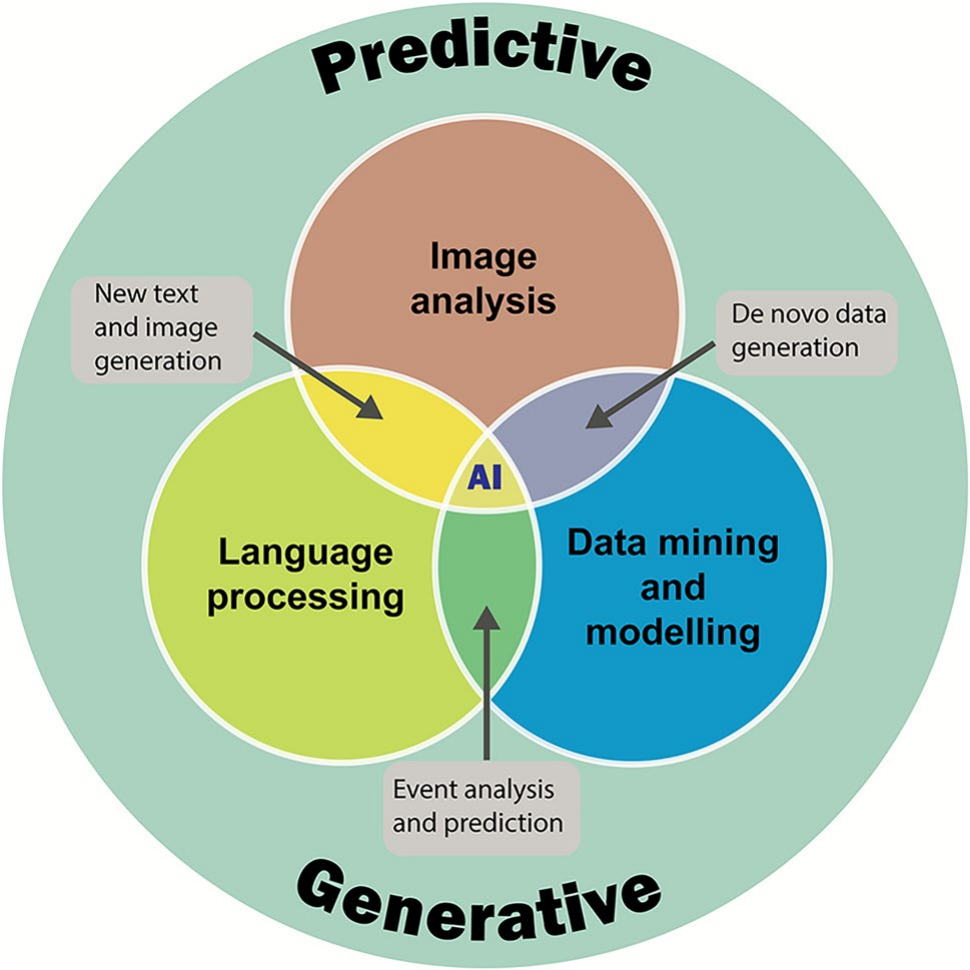
Illustration of the application domains of AI considered in the workshop, each of which can operate in predictive or generative modes

Each can operate in predictive or generative modes, often integrated into hybrid workflows (Fig. 5).

#### Image analysis and cell painting

Several presentations explored the application of predictive AI image analysis, especially classification to cell painting data. The image analysis workflow typically begins with identifying key visual features of the cells and their component parts (nuclei, cytoplasm, organelles, mitochondria etc.), e.g. size, shape, texture, intensity of biomarkers, followed by extraction (i.e. quantified features into numerical representations). Once extracted, the data will be annotated by an expert for model training, which will then be applied to analyse new cell images. The annotated cell feature table can then be used for phenotype identification by training typical machine learning models. These techniques enable the extraction of the difference between cells while filtering out redundant or correlated information. Visualisation of the large feature data for exploration can also be achieved using nonlinear dimension reduction to 2 or 3D dimensions to allow plotting using standard visualisation tools (e.g. Jia et al. (2022)).

##### Imaging layers

The term ‘layers’ refers to predictive modelling that operates to find and analyse objects in an image. For example, this could involve giving the program a set of specific images as a training set that would be the same object but heterogeneous in variables aspects such as colour, position, or size (also called image augmentations). The model would numerically capture this diversity. When applied to a new image, the programme would use this model to find objects that correspond to this model within a landscape of other images. In a cell-based context, two scenarios were presented:

A. The first was within the context of genotoxicity where perturbagens that cause DNA damage can lead to the well-known effect of micronucleus generation. These are well-recognised by microscopists but can be difficult and laborious to find and count. When trained on an appropriate model, an AI image analysis program can be used to find and count these micronuclei even when they are rare in the images (Verma et al. 2018, 2017; Wills et al. 2021).
B. Another example was presented within the context of cell painting. Cell painting is an in vitro assay that relies on the specific staining of cell organelles generating complex cell image changes, which can reflect specific responses of cells to perturbagens. It is being increasingly used for early toxicity assessment (Camilleri et al. 2024; Harte et al. 2024) and proposed as part of the NAMs toolbox (Nyffeler et al. 2020). The image types generated by these methods are well suited for AI-based analysis and they offer a cost-effective way to generate new data, which essentially gives the AI what it needs, a large volume of training data.

#### Natural language processing and large language models

Natural language processing involves using deep learning algorithms to analyse and process structures in text for broad language related tasks. These programs can be used to summarise long pieces of text or find patterns/insights within text, convert from one format to another or extract data and generate new language. Large language models are a subfield of natural language processing models which have been trained on massive amount of text data without task-specific training. Both natural language processing and large language models have applications throughout toxicology such as summarising text across many publications and the extraction of data, for example, for use in a meta-analysis. They can also be used (though this use is not generally supported) in the writing of papers and reports or in the editing support of an author.

**Fig. 6:**
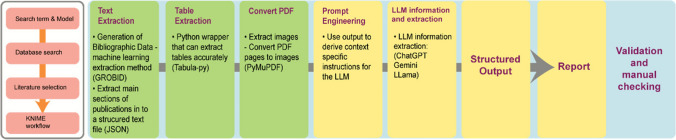
Illustration of a workflow for data extraction from the existing literature, formatting and reporting using AI. [LLM = large language model]

A workflow for the extraction of data and summary of papers is shown in Fig. 6 based on several of the workshop presentations. Both academia, industry and government are using similar workflows to consolidate data from the literature. Shown here is a process that starts with a conventional search term to gather a set of scientific papers in pdf format for analysis into a zip file. From these the text is converted to a human and machine-readable text format (.json). In a parallel or series workflow, the tables in the.pdf files are separately extracted and similarly the images. The text files are then input into an large language models and the prompt file is developed for the machine to learn and then analyse and extract the information that is required from the text files and put this into a structured output ready for report generation prior to human validation and checking (Fig. 6).

#### Data integration & modelling

A process that has much in common with both image analysis and natural language processing is the application of AI to large datasets to find patterns and features. This process uses standard algorithms, such as principal component analysis and hierarchical clustering. The evolution with AI is the use of a training model to refine the processing. The process can operate in a predictive mode to find patterns in a large dataset and use this output in a generative mode as described above for ToxGAN to develop datasets. Applications include multi-omics integration, pattern mining and clustering.

## Regulatory landscape and governance

The integration of AI into regulatory toxicology is already transforming traditional approaches to chemical risk assessment. AI-driven models, particularly those based on machine learning, enable the analysis of complex and high-dimensional datasets, facilitating the prediction of toxicological impacts of chemicals. Applications include predictive toxicology such as QSAR models, high-throughput hazard screening, physiologically based pharmacokinetic (PBPK) modelling and mapping of adverse outcome pathways (AOPs) amongst others. Regulatory agencies are increasingly exploring AI for probabilistic risk assessment, leveraging AI’s ability to provide confidence-weighted predictions.

However, challenges such as data harmonisation, model interpretability, and regulatory acceptance persist (Hartung and Kleinstreuer 2025). Ongoing efforts in explainable AI (xAI) aim to address the ‘black box’ nature of many models to ensure that AI-generated insights are transparent, reproducible, and aligned with regulatory standards. Transparency must be a foundational principle in AI governance. This includes not only the explainability of algorithms but also the traceability and quality of the data used to train and operate these systems. The OECD’s 2024 Revised Recommendation on Artificial Intelligence emphasises that AI actors should commit to transparency and responsible disclosure, providing meaningful information about AI systems, their capabilities, and limitations (Calvino et al. 2025; OECD 2023a; OECD 2024b). When used in chemical risk assessment AI systems must operate within clearly defined ethical, legal, and scientific boundaries. Effective governance ensures that responsibilities and oversight are clearly assigned throughout the AI lifecycle, that systems comply with relevant regulatory standards, and that mechanisms are in place to monitor for unintended consequences such as bias, misuse, or model drift, in an interdisciplinary settings were model developers and regulators collaborate (Wassenaar et al. 2024). This structured, interactive framework is essential to build and maintain trust and accountability in AI-driven decision-making (Hartung et al. 2025b).

AI should serve as a complement to human expertise—not a replacement. Human oversight and interaction remain essential to interpret AI outputs within the appropriate scientific and regulatory context, to ensure ethical decision-making, and to intervene when models produce unexpected or questionable results. This human-in-the-loop approach safeguards the integrity and accountability of AI-driven processes.

Moreover, robust checks and standards must be established to ensure the trustworthiness of data. Poor-quality, biased, or unrepresentative data can lead to harmful outcomes, especially when used in high-stakes domains such as healthcare, criminal justice, or financial services (Hartung et al. 2025a). The OECD’s policy paper on AI, data governance, and privacy highlights the need for integrated approaches that align data governance with AI principles to mitigate such risks (OECD 2024a).

Several key regulatory considerations for the application of AI have been identified (EMA 2024; Mirakhori and Niazi 2025; WHO 2023):

- **Model credibility and validation:** Regulatory use of AI requires robust evidence of model credibility. This includes clear documentation of the model’s development, training data, validation procedures, and performance metrics within its intended context of use;
- **Transparency and explainability:** AI systems used in chemical risk assessment must be interpretable and explainable to regulators, as this is essential for understanding how decisions are made in high-stakes areas such as chemical safety and efficacy. In this regard, technologies that provide uncertainty estimates and define whether a prediction falls within its applicability domain are critical for building trust. These mechanisms are well established in predictive AI through probabilistic modelling and domain applicability checks. However, generative AI models rarely convey uncertainty, for example, by indicating when they lack sufficient knowledge, posing a challenge for their responsible use in regulatory contexts.
- **Data quality, harmonisation and governance**: The reliability of AI outputs depends heavily on the quality, completeness, and representativeness of the input data. Harmonised, well-curated datasets are critical for regulatory acceptance. The increasing availability of public databases and interfaces for their automated import, curation and structuring are critical here (Gao et al. 2025).
- **Risk-based approach:** A risk-based framework is recommended to assess the impact of AI on regulatory decisions. Higher-risk applications (e.g., clinical decision support) require more stringent validation and human oversight;
- **Lifecycle management:** AI models must be monitored and maintained throughout their lifecycle. This includes updating models as new data becomes available and ensuring continued performance over time;

## Ethical and legal compliance

AI applications must comply with existing legal frameworks, including data protection (e.g., general data protection regulation (GDPR) in the EU), and must uphold ethical standards such as fairness, accountability, and non-discrimination.

Ultimately, fostering a human-centric and trustworthy AI ecosystem requires not only technical safeguards but also legal and institutional frameworks that uphold transparency, accountability, and data integrity.

### Lesson learned from the implementation of Omics in regulatory decision-making

The advent of the Omics technologies, particularly transcriptomics, in the late 1990’s and early part of the twenty-first century led to a fundamental change in the ability to monitor changes in biological systems in response to a perturbagen and gave rise to the era of ‘big data’ in biology and toxicology. Big data immediately catalysed challenges relating to data sharing and annotation that sparked the development of FAIR principles, a set of principles that underpin the management, sharing and reuse of big datasets.

At the outset, the thinking and vision behind the application of Omics technologies in chemical hazard assessment was that the simultaneous assessment of multiple biological changes in response to a perturbagen would lead to the ability to detect hazard more efficiently. This was a reasonable vision and desire but has taken a long while to realise. Data storage was addressed relatively early on with the development of databases such as the Gene Expression Omnibus (GEO) and ArrayExpress, which require the inclusion of metadata. With the development of novel multimodal data, in particular images, there will be further data size challenges that will test the limits of databases and transferability, but will likely be overcome by equipment provisions and the ever-increasing capacity of data storage. Experimental metadata is crucial and has been recently addressed via the OECD frameworks for Omics data reporting. Further challenges occur with the multiple ways a big dataset can be transformed and interpreted, involving such changes as background correction, normalisation and statistical analysis. Methods for comparing and reporting data transformations effectively need to be identified. Even 25 years from the advent of genomics these challenges are only now being resolved at the international level, e.g. for Omics (OECD 2023b), and there is substantial work to be done with respect to image analysis and other forms of big data.

The translation to application of Omics in regulatory toxicology for the purpose of product registration is still outstanding. What must therefore be achieved by the international community is rapid development of regulatory structures for AI, learning from the experience with Omics, also recognising that Omics was dependent on technological developments (microarrays to high throughput sequencing for example) that are less of a restriction to the progression of AI.

### FAIR principles: a prerequisite for responsible AI in regulatory science

As AI becomes increasingly embedded in regulatory science and chemical risk assessment, it is imperative that we adopt a principled approach to data stewardship. The FAIR principles—that data should be findable, accessible, interoperable, and reusable—must become a mandatory standard for ensuring the long-term appropriateness, transparency, trustworthiness and the full potential of AI applications.

AI tools rely heavily on high-quality, well-annotated datasets for training and validation. However, several challenges hinder effective data utilisation:

- **Data heterogeneity**: AI must integrate diverse sources such as omics, imaging, and chemistry databases;
- **Storage and transfer**: Large-scale datasets, especially high-resolution images, present logistical and infrastructural burdens;
- **Provenance tracking**: Differentiating between experimental, legacy, and AI-generated synthetic data, and understanding the life cycle of data analysis and model building, is essential for transparency and reproducibility.

Since 2001, Omics research has adhered to metadata standards and raw data deposition practices (e.g., Brazma et al. (2001)). The rise of AI, both predictive and generative, intensifies existing FAIR data management challenges, an issue familiar from Omics application in regulation and research (Gant et al. 2023). There is a pressing need for dedicated facilities or repositories to host training datasets and models. While the scale of AI data may be larger, the foundational principles established for Omics data sharing offer a roadmap for overcoming these hurdles. One of the most promising opportunities lies in reuse of historic in vivo data. These datasets, often underutilised, can serve as critical anchors for training, validating, and contextualising AI models within the framework of NGRA. However, without FAIRification, these data remain fragmented, poorly annotated, and difficult to integrate.

Generative AI introduces new complexities in data transformation and comparison. Generative AI demands reproducibility not only of outcomes but also of the methods used to create synthetic data. Entire datasets generated by AI may resemble test data, yet individual data points often differ. The ability to produce vast quantities of synthetic data raises questions around storage, sharing, and equity, and whether AI-generated data should be treated the same as experimentally derived data.

**Fig. 7:**
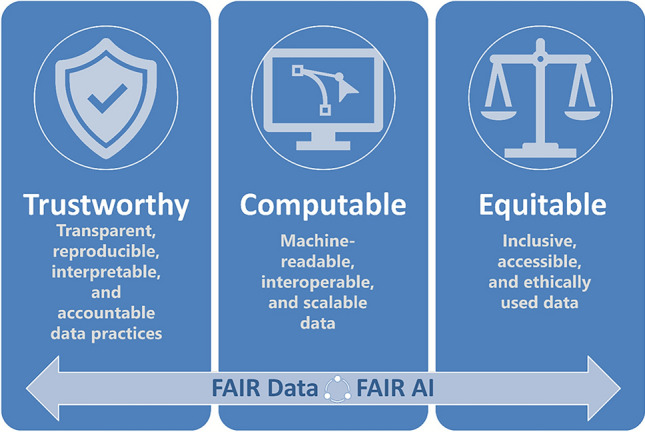
Illustration of the three pillars of FAIR AI

Applying FAIR principles rigorously in AI will lead to more transparent, reproducible, and high-quality outcomes (Fig. 7). In the long term, the benefits of FAIR compliance far outweigh the initial effort required to meet these standards.

To ensure FAIR AI, it is essential for instance to:

- **Label data transparently:** Clearly distinguish between legacy experimental data, newly generated experimental data, and AI-generated synthetic data. This labelling is critical for traceability, reproducibility, and ethical data use.
- **Link AI models to their data sources:** Ensure that AI models are explicitly associated with the datasets they were trained on or used to generate. This supports model validation, reuse, and accountability.
- **Extend metadata standards:** Build on existing frameworks—such as the OECD’s Omics reporting framework (OODRF; OECD (2023b)), EU-ToxRisk, and RISK-HUNT3R—to develop metadata standards tailored to AI-generated datasets. These initiatives offer valuable templates for metadata capture and model annotation. These standards must evolve to capture the unique characteristics and provenance of AI-derived data.
- **Harmonise data models:** Adopt harmonised data models to integrate diverse datasets across Omics, imaging, chemistry, clinical, and exposure science. This enables seamless data interoperability and supports the development of robust, generalisable AI systems.
- **Standardise ontologies:** Use harmonised ontologies—shared, standardised vocabularies that allow us to ‘name things the same way’—to ensure consistent terminology across datasets. Semantic alignment is essential for machine readability, cross-study comparisons, and the development of explainable AI.

Given the rapid pace of AI development, it is critical not to delay the establishment of international standards. Unlike the decades it took for Omics to mature, AI requires immediate and coordinated action to ensure responsible and equitable data use.

The path forward demands strong cooperation and collaboration across sectors, disciplines, and borders. Regulatory agencies, industry stakeholders, academic researchers, clinicians, and data scientists must work together to:

- FAIRify legacy datasets, especially in vivo data;
- Develop and adopt shared ontologies and data standards;
- Create interoperable platforms for data sharing and AI development;
- Foster a culture of openness and trust.

Only through such collective action can we ensure that AI serves as a reliable, ethical, and scientifically sound tool in the future of chemical safety and human health protection.

Overall, the reliability of AI in producing scientifically valid and socially responsible outcomes depends fundamentally on the availability of FAIR data. However, ensuring trustworthiness also requires robust governance frameworks that go beyond data and human oversight. These frameworks must explicitly address AI-specific failure modes—such as adversarial manipulation and recursive model collapse due to synthetic data—as well as human-factor biases that may arise in human oversight. In AI, failure modes refer to specific ways in which an AI system can go wrong or produce unreliable, unsafe, or biased outcomes.

### The influence of new AI legislation on bioscience research

New legislation, such as the EU AI Act^3^ is starting to establish a legal framework for AI use in organisations. These AI regulations play a crucial role in shaping the landscape of AI safety and the advancement of bioscience research by establishing frameworks for ethical application, objective evaluation, risk management, and accountability. These will start to shape the landscape of AI applicability and influence use in chemicals regulation. The EU AI Act has the goal of protecting civil and human rights while promoting innovation, emphasising increased algorithmic transparency, human oversight, and mitigation of AI bias. It also makes exceptions for high-risk AI use in cases of national security. This legislation seeks to foster transparency and establish accountability and reduce the potential for misuse of AI. Such regulatory frameworks must acknowledge and facilitate AI’s beneficial role in accelerating scientific discovery, while also navigating complex implementation challenges related to regulatory harmonisation, effective enforcement, and the inherent technical complexities of AI itself.

The EU AI Act categorizes AI applications based on their risk of causing harm, including “unacceptable,” “high,” “limited,” and “minimal” levels. High-risk applications, like those in healthcare or education, are subject to stringent obligations regarding security, transparency, and quality, and require conformity assessments. The EU AI Act also mandates transparency when AI is involved in content generation and requires documentation of training data. Certain applications with unacceptable risks, such as real-time remote biometric identification in public spaces or social scoring, are outright banned.

## Breakout session insights

Breakout groups on day two of the ECETOC workshop explored opportunities and challenges in applying AI to chemical risk assessment across three key themes: 1) data considerations; 2) identifying possible applications and deployment opportunities of AI and; 3) how to build trust for use of AI in a regulatory context (including lessons learnt from genomics).

### Data considerations

The successful integration of AI into chemical risk assessment is fundamentally contingent upon a single critical prerequisite: the availability of high quality data. As underscored during the ECETOC AI workshop breakout discussions, the foundation of trustworthy and reproducible AI models lies in transparent, high-quality, and FAIR datasets. However, the path toward such a data ecosystem is fraught with technical, legal, organisational, and cultural challenges that require multi-stakeholder collaboration and proactive governance.

Discussion on data considerations was organised into 3 main parts.

#### Part I: Overcoming barriers to data sharing

There are two aspects of data sharing: the landscape of data and data sources, and major barriers.

a. Landscape of Data and Data Sources.

The landscape of data in scientific research is vast and varied, encompassing numerous types of data from diverse sources. This aspect has significant importance in generative AI. These sources include experimental data, observational data, computational data, and more. Each type of data has its own characteristics, formats, and standards, which can pose challenges for effective sharing and integration. Workshop participants identified multiple sources of chemical safety data—including industry-owned repositories, academic databases, federal research resources, and regulatory submissions—many of which are not accessible in formats amenable to AI analysis. For example, the European Chemicals Agency’s IUCLID system, although a rich data source, was flagged as a bottleneck due to its limited machine-readability and inconsistent metadata practices. Similar limitations have been recognised in the literature, with calls for improved accessibility and harmonisation of regulatory databases to facilitate AI-driven toxicology (Rovida et al. 2020).

Understanding the data landscape involves recognising the different origins and types of data, as well as the methodologies used to collect and process them. This knowledge is essential for identifying potential barriers to data sharing and developing strategies to overcome them.

b. Major barriers for data sharing.

Major barriers include concerns over intellectual property and confidential business information, legal and regulatory constraints (e.g., GDPR), technical incompatibility due to lack of standardisation, quality issues and errors in databases and organisational data silos (Fassnacht et al. 2023; Freudenthal et al. 2024; Jussen et al. 2024; Mu et al. 2024; Ponce et al. 2025; Van Panhuis et al. 2014). Companies are often reluctant to share data that confer competitive advantage or risk reputational damage if quality issues are exposed. Moreover, commercial misuse or misinterpretation of open-access data (‘data hijacking’) has been highlighted as a growing concern, especially when public datasets are re-used by commercial actors without sufficient attribution or oversight (Ge et al. 2024; Li et al. 2024b).

Harmonisation of data, which refers to the process of making data compatible and interoperable across different systems and platforms, is a key issue that needs to be addressed alongside the application of FAIR data principles (Freudenthal et al. 2024). This involves standardising data formats, ensuring consistent data quality, and establishing common metadata schemas for data exchange.

Effective data harmonisation is crucial for enabling seamless data sharing and integration. It allows researchers to combine data from multiple sources, facilitating comprehensive analyses and fostering collaboration across disciplines. Harmonisation also enhances the reproducibility of scientific findings, as standardised data can be more easily verified and reused (Aurisano and Fantke 2022; Kush et al. 2020).

To address these barriers, the group proposed several actions:

- **Standardisation:** using OECD Harmonised Templates, controlled vocabularies (e.g., chemical entities of biological interest (ChEBI)), and toxicological ontologies (e.g., OECD Harmonised Templates, AOP-Wiki) to harmonise data from diverse sources;
- **Federated learning models:** and ‘honest broker’ platforms to allow secure data analysis without centralising sensitive datasets (Amankwah and Taylor 2024; Huang et al. 2024; Mammen 2021; Pei et al. 2024; Rieke et al. 2020; Singh et al. 2024);
- **Legal frameworks:** including tiered data-sharing agreements and licensing mechanisms, similar to Creative Commons or the European life-sciences infrastructure for biological information (ELIXIR) European genome-phenome archive (EGA) policies;
- **Positive incentives:** such as demonstrating regulatory acceptability, improving model generalisability, and enhancing public trust in innovation (Mons et al. 2017).

#### Part II: Ensuring data quality and reliability

AI models are only as reliable as the data on which they are trained. Concerns about biased or low-quality input data were frequently raised during the workshop. Errors in labelling, omission of negative results, and lack of context in metadata can result in model misclassification or reduced predictive accuracy (Karimi et al. 2020; Naser 2025). These risks are compounded when synthetic (AI-generated) data is reintroduced into training cycles, leading to the so-called ‘model collapse’ or overfitting to previously seen outputs (Bahov 2025).

A proposed solution was to adopt a tiered data inclusion model: incorporating both GLP (good laboratory practice) and high-quality non-GLP studies, but with weighted trust levels based on validated metadata and reproducibility scores. This is in line with proposals from the AI4Tox and EU-ToxRisk projects, which advocate using multi-source training data while accounting for data origin and quality scores (Moné et al. 2020).

Key factors identified as essential for improving data reliability include:

- Minimum reporting standards and checklists for toxicological data (e.g., ToxRTool, Klimisch criteria);
- Metadata completeness for study reproducibility;
- Access to raw datasets and computational model documentation;
- Tools for AI-assisted quality scoring (e.g., natural language processing-based reviewers or meta-learning algorithms).

#### Part III: Strategies for data curation and lifecycle management

Curation is central to making data AI-ready. Inconsistent units, unstructured formats, missing variables, and poor annotation undermine model development and regulatory credibility. The breakout group members emphasised that data curation should begin at the point of collection—not at the endpoint of submission—embedding FAIR principles from the outset (Wilkinson et al. 2016).

Specific recommendations included:

- Rich annotation using structured formats (e.g., JavaScript object notation (JSON), resource description framework (RDF) data formats), including experimental protocols, compound identifiers, and endpoints;
- Controlled vocabularies and ontologies such as the OECD AOP-Wiki, ChEBI, Observational Medical Outcomes Partnership (OMOP) and eTOX ontology to enable semantic harmonisation (Paini et al. 2022);
- Lifecycle management plans: with version control, access rights, and deprecation policies for outdated datasets, following models from genomics (e.g., US National Center for Biotechnology Information (NCBI), ELIXIR);
- AI for curation: natural language models and unsupervised algorithms can help identify missing metadata, extract structured content from literature, and standardise formats.

Governance challenges—such as who stores and funds long-term data maintenance—mirror those seen in biomedical informatics. National data hubs, industry consortia (e.g., the International Council of Chemical Associations (ICCA) NetChemist), and hybrid funding models were discussed as potential solutions.

### Existing and future applications

Over fifty existing and future applications of AI in chemical risk assessment were identified by the workshop participants. These applications covered seven broad categories:

1. Data and knowledge extraction and synthesis;
2. Data interpretation and analysis;
3. Design of testing, assessment and research strategies;
4. Endpoint prediction;
5. Development and optimisation of software and algorithms for chemical assessment;
6. Chemical monitoring;
7. Chemical management.

Example applications are illustrated in Table 1.

**Table 1:** Example applications for the use of AI in chemical risk assessment that were identified by workshop participants

Application area	Example of application
Data and knowledge extraction and synthesis	Extraction of knowledge for a systematic review; Extraction of data for model development
Data interpretation and analysis	Analysis of histological data; Identification of data gaps for different classes of chemicals; Quality scoring of data; Dealing with uncertainty
Design of testing, assessment and research strategies	Development of a testing and modelling strategy to support the risk assessment of a new chemical; Development of new improved guidance based on the overall knowledge base
Endpoint prediction	Prediction of physico-chemical properties; Prediction of ADME properties of a new molecule; Prediction of toxicological effects of a new molecule; Mixture assessment; Interspecies extrapolation; In vitro to in vivo extrapolation
Development of software for chemical assessment	Development of new exposure, PBPK, and effect models; Optimisation of an exposure model; NGRA Toolbox
Chemical monitoring	Post-authorisation monitoring of drug side effects; Interpretation of human biomonitoring data; Near real time detection of a chemical incident in a city
Chemical management	Chemical incident management; Design of safer chemicals; Design of safer synthesis processes

**Fig. 8:**
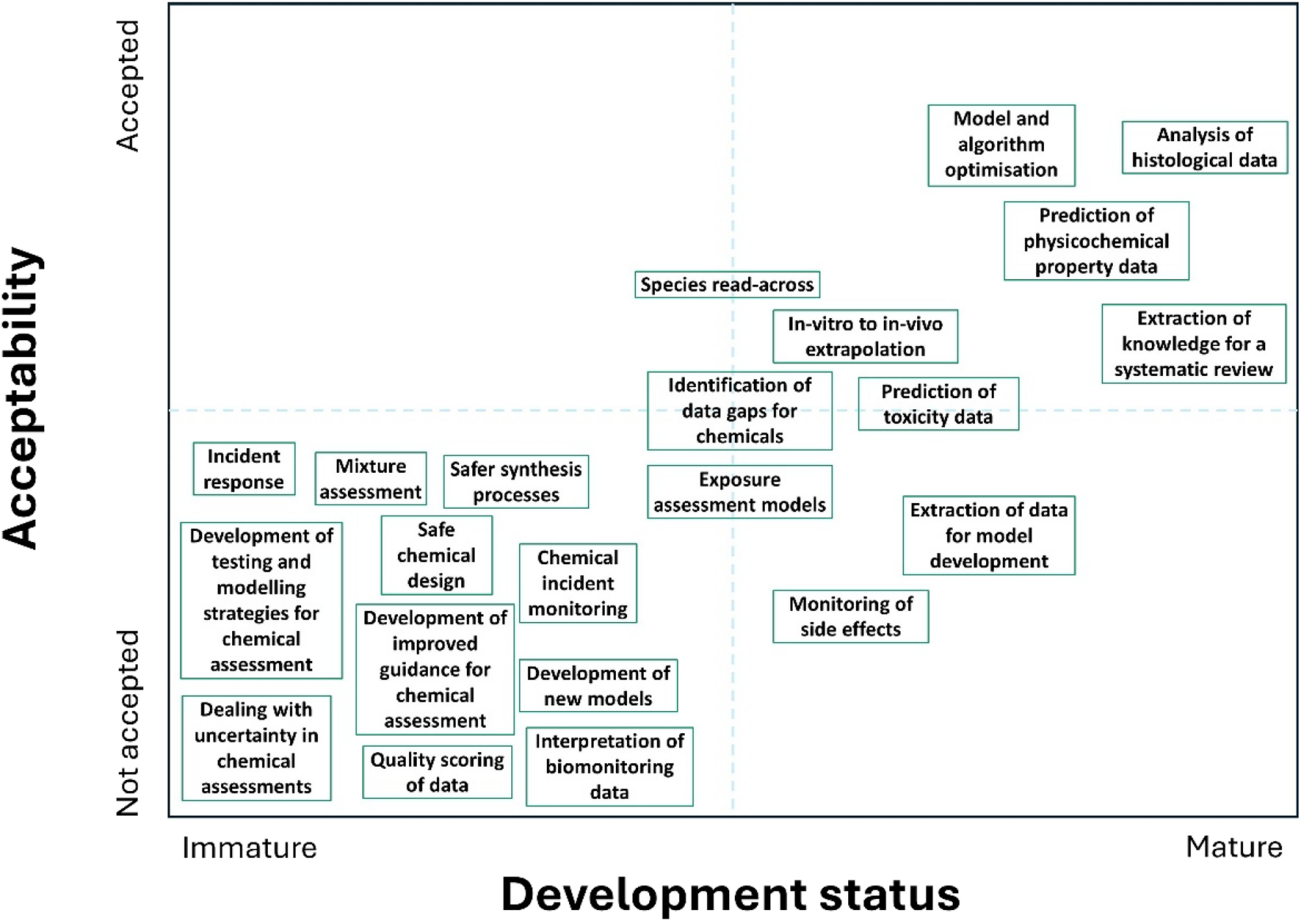
Matrix indicating the development status and acceptability of the different applications of AI, collected opinions of the workshop participants, to support chemical risk assessment

For each application, participants assessed the application in terms of the maturity of the AI approach, the reliability of the approach and the acceptability of the results to users, such as regulatory agencies. Only a few applications, such as the use of AI to support systematic reviews, the prediction of the physico-chemical properties of a substance, the analysis of histological images, and optimisation of algorithms and models were deemed to be at a mature level of development, and to be reliable and acceptable. Approaches for predicting (eco)toxicological endpoints and for read-across were deemed to be still work-in-progress with moderate acceptability. Many applications were considered to be at a low level of maturity and to have potentially low reliability and acceptability for regulatory decision-making, while also representing the highest priority opportunities for investment, characterization, and further development (Fig. 8).

The results of this exercise show that AI has great potential to support chemical risk assessment but at the current time, this potential is not being fully exploited.

### Building trust

Trust is a complex matter that has different perceptions. Trust must be evidenced, not asserted. For AI, trust will require a demonstration that the AI tool has been used responsibly, is reliable and has been used transparently and ethically. At all levels, trust can be built by showing that AI can assist in the removal of human bias and derive greater understanding from a dataset than would be possible from non-AI approaches. For this to occur, there needs to be standards, methods, policies, and principles developed, adopted, and adhered to by all those using AI tools in the analysis or generation of data. Further, in the problem formulation preceding a regulatory study, there should be a clear statement of benefit for the application of AI in the study and this should include an analysis of the check points and technical limitations. The exploration of building trust, and the tools needed to do this, should be an ongoing debate with both the regulators and the public leading to revisions, as necessary, as the AI technology develops.

**Fig. 9:**
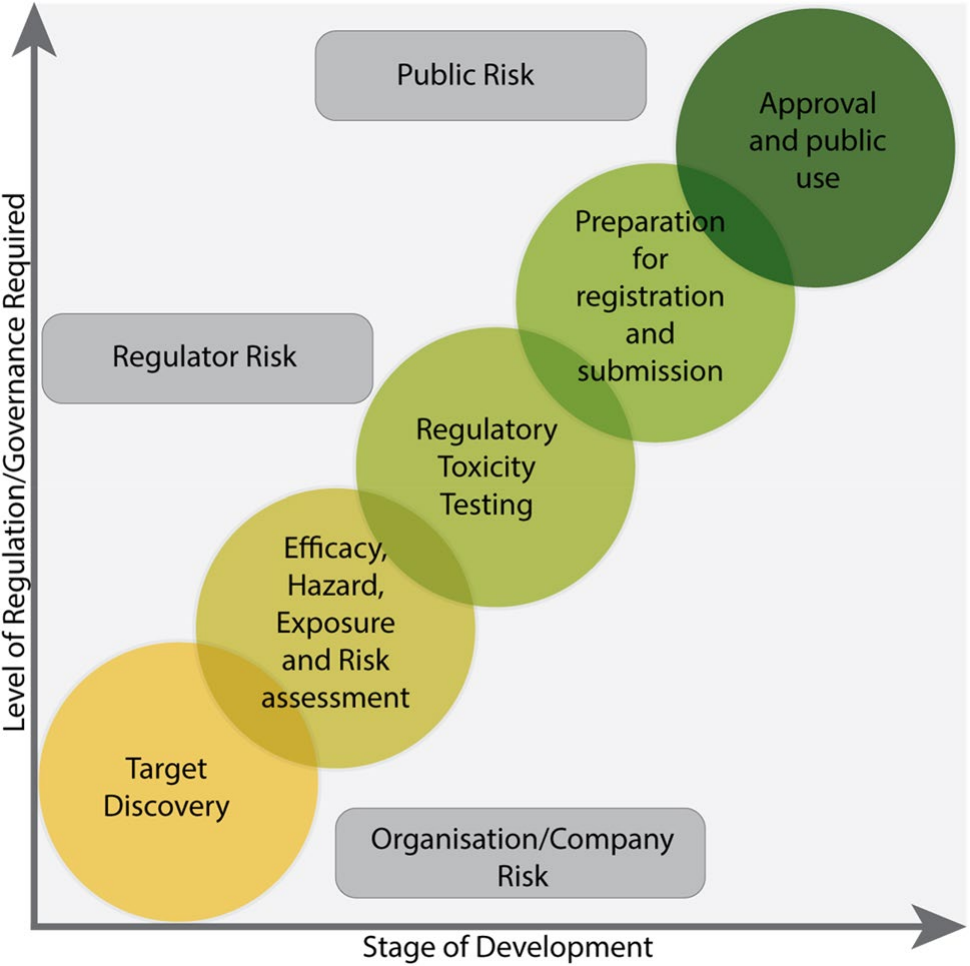
Plot illustrating the level of risk governance required with application of AI at different levels of the regulatory process, the level of risk and to whom

For AI applications in chemical regulations, the breakout group members considered that it could be condensed to a problem statement of whether the output of AI could be trusted even when the complexities of the model or the programme are likely not to be fully understood perhaps by the operator and to a larger extent the regulator. This then led to consideration of how to build trust and in particular what level of governance is required for this trust, which is going to be dependent on the way in which the technology is being used (Fig. 9). If, for example, AI is being used within a company for leading chemical selection or early-stage activity or toxicity screening, then there is little or no external governance required because any failure in the AI is only going to impact the user or the company. In this case, the company decides on the cost of failure and applies its own internal governance. However, a different level of trust and associated governance is required when there is an impact on a third party, such as the public. A third party has no influence over the AI being used (but full responsibility on the decisions taken based on the results obtained using the AI) and therefore the outputs must be entirely trustworthy.

During the workshop, the breakout group’s discussion of this scenario circled back to the FAIR principles. Whenever a third party is impacted by the application of AI, FAIR principles should be applied to minimize the level of risk. This includes ensuring that the training data used in the building of models, the programs and, where applicable, test data along with a full declaration of the process are FAIR, and allow others to assess and check the process and data with full transparency. Benchmarking is one way forward, where there is one set of analysis procedures that is always followed, against which other methods of data transformation can be compared.

However, predictive and generative AI, however, pose different challenges in terms of building trust. Benchmarking can probably be used effectively in predictive AI. For example, if the output from control samples does not match the expectation, then clearly the model and/or algorithms being used need to be revisited. For generative AI, new data is being generated that is based on experimental training data but not derived from experimental procedures. In one respect, such data is already benchmarked in that it is compared to a test dataset derived from the original experimental data as part of the procedure (Fig. 3). Thus, in effect, these data cannot be differentiated from the training data. If these data are therefore used in a way where there could be an effect on a third party, such as the public, when used in support of a regulatory submission, there is a breach of trust if this is not fully declared. It is not even sufficient in this scenario to demand the inclusion of metadata as a means of verifying the dataset, because there is the possibility that this could also have been derived from a generative synthetic AI procedure. Adherence to GLP was identified as the way forward, whereby some of the record keeping would verify that the dataset is experimental; or not, as appropriate.

In conclusion, the group considered four principles necessary to develop trust in the application and use of AI in a regulatory context:

1. **Transparency and data availability**: The models, data and methods used need to adhere to FAIR principles and be available and accessible without cost. Where standard protocols exist, these should be adhered to. Where a benchmark has been used, the standard/control data used for the benchmarking should be similarly available. The tests used to ascertain the quality of the benchmarking should also be available. Where generative AI has been used to derive a dataset, this needs to be declared and both the training and test data made available. Reporting frameworks, such as those now developed for Omics (OECD 2023b) should be developed to ensure all the relevant information to aid transparency is captured and reported.
2. **Trust and human evaluation:** Trust can be clearly jeopardised by AI, and this has been clear in other areas such as the DeepFake models. To ensure this does not happen for the applications of AI in chemical risk assessment there is a need to consider the AI as Augmented Intelligence, as introduced earlier. AI can assist in the processing and generation of data but needs to be applied in an open and transparent manner and the output is always subject to human evaluation. If this principle is adhered to, then trust will be maintained and developed. Thus, there is a role and will continue to be a role for humans (Biological Intelligence) in the application of AI.
3. **Communication**: This is linked to transparency and data availability. Where AI has been used it should be declared and, as above, so should all associated methods and data. It could also be useful in communication to have a form of problem formulation that would underpin why AI was used and the benefits to its application to these data and any issues that are encountered.
4. **Ethical issues**: Particularly for Generative AI, where AI-generated synthetic data could be presented as new experimental data, for example the use in the Deep Fake scenario, then there is a clear need for transparency and communication. Consideration should be given to whether such data crosses boundaries of being ethically acceptable, and these concerns should be recorded for transparency. For example, an AI-generated epidemiological dataset might by chance have person-specific data that could also match to real individuals and cause alarm or distress.
  These principles should be developed now, even if they are imperfect or require future modification as the technology develops. The alternative is that an atmosphere of mistrust is allowed to be built, which will undermine the advantages that can be gained from the application of AI in chemical risk assessment.
  There is therefore a need for reporting frameworks and standards in all the above areas where AI-generated data is being used in the public domain or could affect a third party (Fig. 9). AI analysis and AI-generated data should only be accepted from those that have adhered to these principles and thus can be assured to have acted responsibility. There will be a need for such responsible use to the monitored by an accepted third-party body.

## Conclusions and recommendations

The key takeaways from the workshop presentations and breakout groups discussions are as follows:

- AI enhances but does not currently replace human judgment in risk assessment;
- Generative and predictive AI offer complementary functions in toxicology;
- FAIR data principles are critical for transparency and reproducibility;
- Governance frameworks must evolve rapidly to keep pace with AI development, particularly for generative models;
- Lessons from Omics data analysis and regulatory acceptance can inform responsible AI integration and acceptance;
- Harmonised international frameworks are urgently needed to support development, validation, transparency, and trust in AI applications.

The clear message from this workshop is that AI has already become mainstream and will be an integral part of the future of chemical risk assessment. It is not a question of considering how to apply something that is under development, but rather how to make best use of AI while ensuring transparency, ethical use and application of FAIR principles to a tool that is already here. AI represents a major advancement on existing tools, in that it is usable across a spectrum of toxicological science activities, from literature reviews to chemical selection, hazard identification, exposure assessment and report writing. Furthermore, AI works not only with existing data but has the power to derive new data and works equally well with numbers, images or text.

When Omics first became available about 25 years ago there was a similar excitement for what could be achieved with this new tool. We are now seeing the same with AI. Comparing AI with Omics shows that the scope of applicability with Omics is substantially less than with AI. Omics, while offering a wider view of changes in the biological system in response to a perturbagen, only works with numerical data (changes in mRNA transcript level, metabolite, DNA sequence etc.); AI, instead, deals with all forms of multimodal data. Perhaps fundamentally, Omics works only in conjunction with a biological system, it is a means for measuring changes or cataloguing biological status at a point in time (e.g., DNA sequencing). In addition, AI can work with experimental data or generate its own new synthetic data. This raises opportunities and challenges.

The opportunities of AI application in chemical risk assessment presented in this manuscript are many, and include:

- Extraction and consolidation of existing knowledge more easily, quickly and concisely;
- Analysis of experimental data like Omics data or images more accurately and far faster than is possible for a human;
- Data consolidation and even generation of new synthetic data in the absence of an experimental system.

The challenges, and potential solutions, are several and include:

**Accuracy**

While conventional data analysis relied on fixed algorithms whose output depended entirely on the input data, for AI the output is dependent on the training data and the input data. If the training model is not sufficiently robust, or has been in any way contaminated by data that are not relevant or accurate in respect of the system under analysis, then the output will not be meaningful. There is a well-established concept, that traces its origins to the start of computing, that has several variations but condenses to ‘trash in trash out’.

**Transparency**

**Fig. 10:**
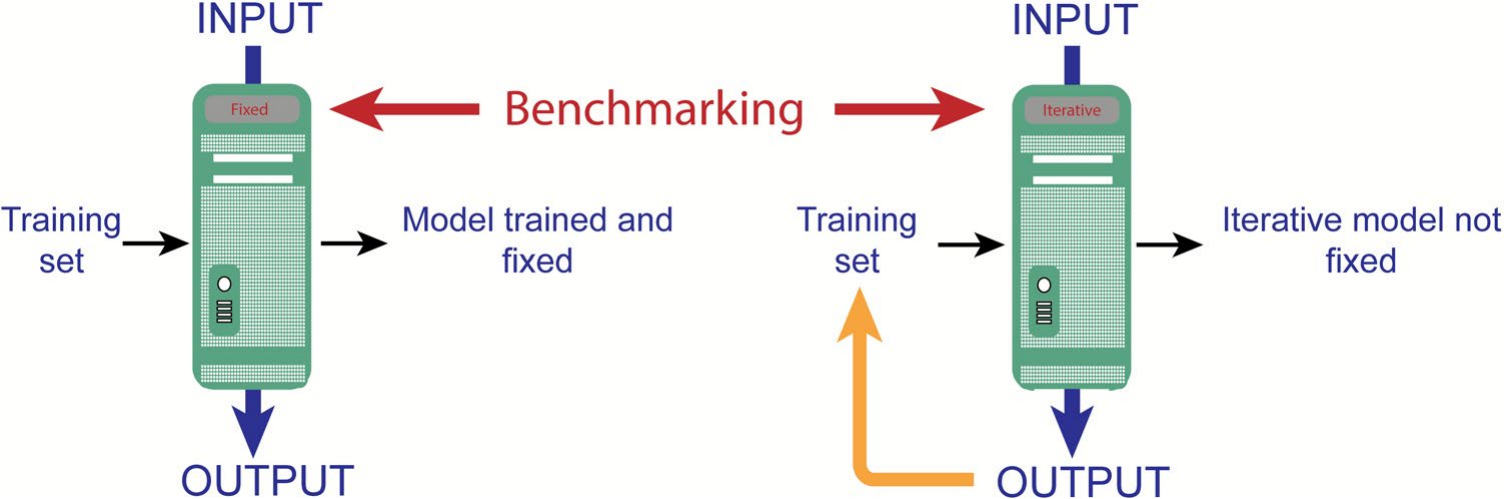
Schematic illustration of the use of positive and negative controls for quality control of output from an AI model

Given that the output from an AI analysis is dependent on the training model used, in the governance of these methods, particularly where the output is relevant to a third party such as the public, there is a need for the training to be transparent. This might be easier to articulate than to achieve. Where output data is continually added into the training model to improve performance, the model data is not a static entity. One way of controlling this could be to rely on simple experimental principles such as the inclusion of positive and negative controls for which the outcome is clearly known. If these do not produce the required output from an AI analysis, then clearly the training model or the algorithms in use are not appropriate for the analysis that is being conducted (Fig. 10).

**Use of generative AI**

Generative AI, i.e. that which produces new data using a model in the absence of an experimental system, provides further challenges. First there is the issue of distinguishing AI-generated synthetic data from new experimental data. It is not inconceivable to consider that AI synthetic data could be used to support a product registration for which an experimental study has not actually been undertaken. Given that all the data for an experiment, including the metadata, could be generated in this manner, this clearly provides significant challenges in terms of governance. The principles of good laboratory practice (GLP) could provide a means for ensuring that data submitted in support of a product is generated from an experimental system rather than AI, but the GLP procedures need to be reviewed in the face of the AI challenge. This is not to suggest that data from generative AI does not have a place in regulation. Where data generated by AI can contribute to the reduction, refinement and replacement of animals in hazard assessment there is clearly a role for generative AI. Such data needs to be clearly distinguished, and the model and algorithms made available and quality-controlled. This latter challenge again could potentially be achieved by consideration of the principles of good scientific endeavour with the use of positive and negative controls.

**Data governance**

Lastly, as considered above, there are the issues of FAIR principles with AI data. All data, including the models and algorithms used to generate it, should always be available when the output is in the public domain. Given the volumes of data that can be generated, the applicability of AI to all forms of data and the speed of current development, this is going to be challenging. This is a challenge that needs to be met to ensure that confidence in AI is not lost and to ensure that there is a more rapid translation into use in the regulatory domains than we have seen with Omics technologies. If underlying trust in the data, its generation or transformation is lost, then the process of transfer from research to regulation is hindered and may take many years to resolve, as it has been the case with Omics technologies. It is therefore important to learn from the experience with Omics and start the process of developing frameworks for the application of AI in regulation now, recognising that the reporting frameworks will need to evolve as the technology and the understanding of the application domain for that technology develops.

The use of AI in regulatory toxicology is not a matter of choice, it will happen regardless of what we decide. The objective should be to make AI work well in regulatory toxicology, not only because of the benefits this technology offers, but also because its implementation is already advancing at a tremendous pace. Application and trust building will not be trivial issues, and overcoming the challenges highlighted in this workshop will require significant efforts from all sectors.

## Footnotes

1 This article concentrates on the value of a trusted and responsible AI assisting and augmenting human-based risk assessment in the near term.

2 https://ntp.niehs.nih.gov/data/drugmatrix

3 https://eur-lex.europa.eu/legal-content/EN/TXT/?uri=CELEX:32024R1689
